# A homeopathic remedy from arnica, marigold, St. John’s wort and comfrey accelerates *in vitro* wound scratch closure of NIH 3T3 fibroblasts

**DOI:** 10.1186/1472-6882-12-100

**Published:** 2012-07-18

**Authors:** Katarina Hostanska, Matthias Rostock, Joerg Melzer, Stephan Baumgartner, Reinhard Saller

**Affiliations:** 1 Institute for Complementary Medicine, University Hospital Zurich, Raemistrasse 100, Zurich, 8091, Switzerland; 2 University Medical Center Hamburg-Eppendorf, University Cancer Center Hamburg, Hubertus Wald Tumor Center, Martinistrasse 52, Hamburg, 20246, Germany; 3 Institute of Complementary Medicine KIKOM, University of Bern, Imhoof-Pavillon, Insel-Spital, Bern, 3010, Switzerland; 4 Center for Integrative Medicine, University of Witten/Herdecke, Gerhard-Kienle-Weg 4, Herdecke, 58313, Germany; 5 Society for Cancer Research, Kirschweg 9, Arlesheim, 4144, Switzerland

**Keywords:** Wound healing, 3T3 fibroblasts, Homeopathic remedy, *Arnica*, *Calendula*, *Hypericum*, *Symphytum*

## Abstract

**Background:**

Drugs of plant origin such as *Arnica montana*, *Calendula officinalis* or *Hypericum perforatum* have been frequently used to promote wound healing. While their effect on wound healing using preparations at pharmacological concentrations was supported by several *in vitro* and clinical studies, investigations of herbal homeopathic remedies on wound healing process are rare. The objective of this study was to investigate the effect of a commercial low potency homeopathic remedy Similasan® Arnica plus Spray on wound closure in a controlled, blind trial *in vitro*.

**Methods:**

We investigated the effect of an ethanolic preparation composed of equal parts of *Arnica montana* 4x, *Calendula officinalis* 4x, *Hypericum perforatum* 4x and *Symphytum officinale* 6x (0712–2), its succussed hydroalcoholic solvent (0712–1) and unsuccussed solvent (0712–3) on NIH 3T3 fibroblasts. Cell viability was determined by WST-1 assay, cell growth using BrdU uptake, cell migration by chemotaxis assay and wound closure by CytoSelect ™Wound Healing Assay Kit which generated a defined “wound field”. All assays were performed in three independent controlled experiments.

**Results:**

None of the three substances affected cell viability and none showed a stimulating effect on cell proliferation. Preparation (0712–2) exerted a stimulating effect on fibroblast migration (31.9%) vs 14.7% with succussed solvent (0712–1) at 1:100 dilutions (p < 0.001). Unsuccussed solvent (0712–3) had no influence on cell migration (6.3%; p > 0.05). Preparation (0712–2) at a dilution of 1:100 promoted *in vitro* wound closure by 59.5% and differed significantly (p < 0.001) from succussed solvent (0712–1), which caused 22.1% wound closure.

**Conclusion:**

Results of this study showed that the low potency homeopathic remedy (0712–2) exerted *in vitro* wound closure potential in NIH 3T3 fibroblasts. This effect resulted from stimulation of fibroblasts motility rather than of their mitosis.

## Background

Wound healing plays a central role for the physical health of the human being. The search for wound healing agents is one of the oldest challenges in medicine, as the mechanism involved in the repair of damaged tissue is yet not fully understood. Skin wound healing is a dynamic process in which different cell types, such as fibroblasts, leukocytes, monocytes/tissue macrophages as well as endothelial and epidermal cells cooperate to restore the affected skin. This highly coordinated process includes a series of both simultaneous and overlapping phases which promote an efficient healing [1,2].

Since ancient times herbal medicines have been widely used all over the world and have been well recognized by the physicians and patients for their therapeutic value.

Various extracts from numerous plants that have been used in wound care, including traditional European plants such as arnica, marigold and St. John’s wort have been reported to accelerate the wound healing process [3-10]. However, in these studies herbal preparations at pharmacological concentrations were used in humans as well as in animals or in *in vitro* experiments.

Homeopathy is a therapeutic method based on the empiric law of similars with the hypothesis, that a given substance can cure in a diseased person the symptoms that it produces or causes in a healthy person [11]. There are some contradictory results regarding the effect of homeopathic remedies in low concentrations on wound healing. In several animal and human studies a wound healing activity has been observed [12-15]. On the other side no effect could be found in other trials [11,16,17]. *In vitro* studies on the wound healing of remedies at homeopathic dilutions are scarce [18].

Therefore, the objective of our study was to evaluate, through an *in vitro* model in blinded manner, the efficacy of a commercial homeopathic remedy, Similasan® Arnica plus Spray consisting of arnica, marigold, St. John’s wort and comfrey. It is used to treat injuries such as sprains, bruises, contusions, haematomas, muscle soreness or pain following operations and bone fractures. We used the well-established *in vitro* scratch assay in mouse NIH 3T3 fibroblasts, that mimics the behaviour of these cells during migration *in vivo* and is compatible with microscopy and cell imaging software [8,19].

## Methods

### Preparation of Similasan® Arnica plus Spray and controls

Similasan® Arnica plus Spray is an over-the-counter homeopathic preparation composed of extracts of four plants that have been moderately diluted. The potency level of individual components is 4x (arnica, St. John’s wort, marigold) and 6x (comfrey). The homeopathic preparation was potentized (diluted in the ratio 1:10) at Similasan AG (Jonen, Switzerland), starting from the single alcoholic potencies arnica 1x (*Arnica montana* L*.*)*,* marigold 1x (*Calendula officinalis* L*.*), St. John’s wort 1x (*Hypericum perforatum* L*.*) and comfrey 3x (*Symphytum officinale* L.) manufactured by Herbamed (Buehler, Switzerland), following the German Homoeopathic Pharmacopoeia (GHP1) [20] and corresponding descriptions 4a for arnica, 3a for marigold and St. John’s wort, and 2a for comfrey. The four components were potentized separately up to 3x (*Arnica, Calendula, Hypericum*) and 5x (*Symphytum*), respectively. The last potentization step was performed with all four components combined at equal quantities. Similasan® Arnica plus Spray (lot number 10079) contained 2.80 mg *Arnica*, 0.279 mg *Calendula*, 0.864 mg *Hypericum* and 7.95 μg *Symphytum* dry herbs in 100 g remedy. In all assays comparison was made between solvent (0712–1) serially succussed as was done with the active remedy (0712–2) but without the initial addition of single components. Unsuccussed control containing 22% alcoholic solution in distilled water (0712–3) was also used in the present study.

### Blinding procedure

Both homeopathic remedy and succussed placebo at volume of 20 ml in twenty dark glass ampoules were coded by the producer 0712–2 (1–20) and 0712–1 (1–20), respectively. Study was designed in double - blind manner. Blinded investigators re-coded the ampoules again. Unsuccussed solvent (0712–3) was unblinded. Three independent sets of experiments were performed for each assay.

### Cell line and culture conditions

Mouse NIH 3T3 fibroblasts (ATCC, Rockville, USA) were kindly supplied by Dr. E. Fassler (University of Applied Sciences Northwestern Switzerland, Muttenz, Switzerland) and cultured in Dulbecco’s modified Eagle’s medium (DMEM) supplemented with 5% fetal calf serum (FCS), 4 mM L – glutamine, 1% penicillin/streptomycin under a fully humidified atmosphere containing 5% CO_2_ at 37°C. For experiments, cells were collected from subconfluent monolayers with trypsin/EDTA. Cell viability was higher as 95% using trypan blue dye exclusion staining. The studies were carried out using cells from passages 3 – 8 in DMEM medium containing 2% FCS. In all experiments untreated cells were used as negative controls. All cell culture reagents and recombinant human epidermal growth factor (EGF) used as positive control in the chemotaxis migration assay were obtained from Sigma (Buchs, Switzerland).

### WST-1 cell viability assay

The effect of substances 0712–1, 0712–2 and 0712–3 on the viability of NIH 3T3 cells was determined after 24 and 48 h treatment using WST-1 assay as previously described [21]. Briefly, NIH 3T3 cells were dispensed in 96-well flat-bottomed microtiter plates at a density of 1 × 10^4^ cells/well and incubated with tested substances at 1/10, 1/100 and 1/1000 dilutions for 20 h and 44 h followed for 4 h with a tetrazolium salt WST-1 (4-[3-(4-iodophenyl)-2-(4-nitrophenyl)-2 H-5-tetrazolio]-1,3-benzene disulfonate) from Roche Diagnostica (Rotkreuz, Switzerland). The cleavage of WST-1 to formazan by metabolically active cells was quantified by scanning the plates at 450 nm and 650 nm reference wavelength in a microtiter plate reader. Test medium was used as background control. Three independent sets of experiments performed in triplicates were evaluated. The effect of vehicle ethanol on the NIH 3T3 cell viability at concentration of 0.5% and 1% was tested in parallel. Viability of treated cells was normalized to the untreated control cells.

### 5-Bromo-2-deoxyuridine (BrdU) incorporation

NIH 3T3 cells were precultured for two days in DMEM medium supplemented with 1% FCS and then seeded at a density of 1 × 10^4^ into a 96 wells microtiter plate and cultured in the test medium (DMEM without FCS) in a presence or absence of tested substances for 48 h. As positive control DMEM with 5% FCS was used. Solvent ethanol (0.5% v/v) was tested in parallel. BrdU is a DNA specific analog of ^3^ H] thymidine. Therefore for the quantification of cell proliferation the BrdU Cell Proliferation Assay from Oncogene Research Products (San Diego, USA) a non-isotopic enzyme immunoassay [22] was used according to manufacturer’s instructions. In brief, during the final 24 h of culture 10 μM BrdU was added to the wells and BrdU was incorporated into DNA of dividing cells. BrdU incorporation was then evaluated by measuring the absorbance at 450–540 nm according to the manufacturer’s protocol. Experiments in triplicate repeated three times were evaluated. Two types of controls, only culture medium as blank and wells with unlabelled cells as background were also set.

### Transwell chamber migration assay

To investigate the migration of cells we used the most commonly applied *in vitro* assay, namely the transwell chamber assay using culture inserts with a 8 μm pore-size filter barrier from BD Biosciences (Bedford, USA) [23]. NIH 3T3 cell suspensions (3x10^4^ cells/filter) with or without substances 0712–1, 0712–2 and 0712–3 at 1/10, 1/100 and 1/1000 dilutions were added to the upper compartment whereas the bottom wells were immediately filled with conditioned medium (10% FCS) of fibroblasts as chemoattractant. As positive control EGF (2 ng/ml) was used. After 24 h of incubation, the non-migrated cells in the upper chamber were gently scraped, and the adherent cells present on the lower surface of the insert were fixed and stained with 0.5% crystal violet in 20% methanol. Quantification of migrated cells was determined after extraction of adhesive cells with 30% acetic acid and the absorbance of the cell lysate was scanned by a microplate reader at 540 nm. Each migration experiment was carried out in duplicate and repeated three times. Data are expressed as percent of migration through the cell culture inserts relative to the untreated controls.

### *In vitro* wound healing (scratch) assay

The effect of substances 0712–1, 0712–2 and 0712–3 at 1/100 and 1/1000 dilutions on wound closure was investigated with CytoSelect ™Wound Healing Assay Kit (Cell Biolabs, Inc., San Diego, USA). NIH 3T3 fibroblasts (25 × 10^3^/500 μl) in DMEM containing 5% FCS were seeded into 24-wells tissue culture plate containing proprietary treated inserts in the plate wells with their “wound field” aligned in the same direction and incubated for 24 h to allow the cells adhere and reach the 60-80% confluence. After removing the inserts from the wells the medium was carefully aspired and wells were washed with test medium (DMEM containing 2% FCS) to remove dead cells and debris. Finally, the cells were treated with different concentrations of tested compounds for further 24 hours. Migration into the wound field was determined by using manual fixing with cell stain solution according to manufacturer’s instructions. Representative images focused on the center of the wound field were photographed. Microscopic imaging of wound closure was analysed using CellD software [24]. Three sets of experiments in duplicates were performed. The influence of compounds on wound closure was compared to untreated control. As positive control DMEM with 5% FCS was used. Density of cells in wells without created wound area (confluent area) was used as 100% wound closure.

Experiments were evaluated using following formula:

Wound closure (%) = [(test compound (%) – untreated control (%))/ (confluent area (%) – untreated control (%))] × 100

### Statistical analyses

For each parameter, average values with standard deviations (mean ± SD) were calculated. Transwell migration assay data were analysed by a two-way ANOVA with the independent factors experiment (1–3) and treatment (n = 11 parameters) followed by Bonferroni post-hoc tests. Monolayer wound healing assay data were analysed by a two-way ANOVA with the independent factors experiment (1–3) and treatment (n = 6 parameters) followed by Bonferroni post-hoc tests. Differences were considered significant if p < 0.05. Statistical analysis was performed with Statistica 6.0 (Statsoft Inc., Tulsa, USA).

## Results and discussion

### Cell viability and proliferation response

Cell-based assays can be influenced by cytotoxic effects resulting in false negative results. Therefore, the effects of substances 0712–1, 0712–1 and 0712–3 on NIH 3T3 cell viability were studied. For the assessment of cell survival the WST-1 assay was used, which measures the dehydrogenase activity of viable cells by the cleavage of the tetrazolium salt to formazan in viable cells. Because of possible interference of natural substances with another tetrazolium salt MTT, we first measured the direct reductive potential of all substances in a cell-free system. None of them differred from the blank (medium only). Absorbance values (450–650 nm) for substances were between 0.102-0.113 in comparison to 0.111 of blank value. Cell survival was estimated after 24 h and 48 h treatment according to the following criteria. Cultures with more than 90% viable cells were considered to be unaffected, 80 – 90% as modestly affected, and values of less than 80% viable cells were ascribed to cytotoxic effects of the compound. Considering the above-mentioned criteria, no cytotoxicity of substances was observed. Substances 0712–1 (succussed solvent) and 0712–2 (remedy) exerted a modest effect at the 1:10 dilution, which could be related to the concentration of ethanol of about 2% at this dilution level (Table 1). The vehicle controls at concentrations of 0.5% and 1% did not affect the viability of NIH 3T3 cells. Cell survival was higher than 95% at both concentrations. Usually ethanol concentration up to 1 - 2% does not affect the survival of most cell lines. However, the cytotoxicity of ethanol on different cell cultures at higher concentration is well known [25]. Survival of cells after 48 h culture was equal to survival after 24 h (Table 1). The 48 h cell viability was estimated also, because the effect of substances on the cell proliferation was measured after two days incubation.

**Table 1:** **Effect of substances on NIH** 3T3 **cell viability and cell growth**

**Substance**	**Dilution**	**Cell survival [%]**		**Cell growth [%]**
		**24h**	**48h**	**48h**
0712-1	1/10	83.3 ± 1.2		83.0 ± 2.7
0712-1	1/100	92.3 ± 0.6	92.3 ± 3.2	86.7 ± 2.5
0712-1	1/1000	98.7 ± 1.5	95.0 ± 2.7	93.7 ± 0.6
0712-2	1/10	83.0 ± 1.0	82.3 ± 0.6	81.3 ± 2.1
0712-2	1/100	92.7 ± 3.8	93.0± 0.2	90.7 ± 3.2
0712-2	1/1000	93.3 ± 1.2	95.7 ± 1.2	96.0 ± 1.0
0712-3	1/10	97.0 ± 3.0	93.3 ± 0.6	91.3 ± 1.5
0712-3	1/100	99.3 ± 4.9	96.0 ± 2.0	94.0 ± 2.0
0712-3	1/1000	103.7 ± 2.1	99.7 ± 1.2	95.0 ± 2.0
pos.ctrl	DMEM	n.d.	n.d.	140.5 ± 9.5
	5% FCS			

Exerted effects of substances were standardized to untreated controls. n.d.- not detected. Results are presented as average ± SD from three independent experiments performed in triplicates. Cell survival was assessed with the WST-1 assay, cell growth with the BrdU assay.

In living humans and animals, the wound healing process includes the following phases: blood coagulation, inflammation, cell proliferation, cell migration, lesion contraction, and remodelling. All these phases overlap to promote efficient healing [2]. At first, we chose to carry out the proliferative effect of substances on NIH 3T3 fibroblasts. The proliferative response was based on a quantitative analysis of the percentage of cells staining positive for BrdU incorporation. As positive control DMEM with 5% FCS was used, which caused a proliferation stimulation of 40.5 ± 9.5%. Absorbance value for the positive control was 1.793 ± 0.23 in comparison to 1.277 ± 0.15 for the negative control. The levels of proliferation found in response to substances 0712–1 and 0712–2 were compared to those found using 0% FCS as negative control. No proliferation effect could be found with both substances for the cells at any concentration (Table 1). Growth of cells was modestly reduced by about 10% and by about 5% at 1:100 and 1:1000 dilutions of both substances, respectively. These results could be in accordance with findings, that the high level of cellular confluence down-regulates proliferation [26]. The down-regulation of about 17% (1.06 absorbance value) of the cells was found at the dilutions of 1:10, and could be ascribed to the effect of 2% ethanol as shown by the survival experiments. The results of cells survival by measuring dehydrogenase activity in viable cells correlated well with the BrdU incorporation into DNA of dividing cells.

### Chemotactic migration response

**Figure 1:**
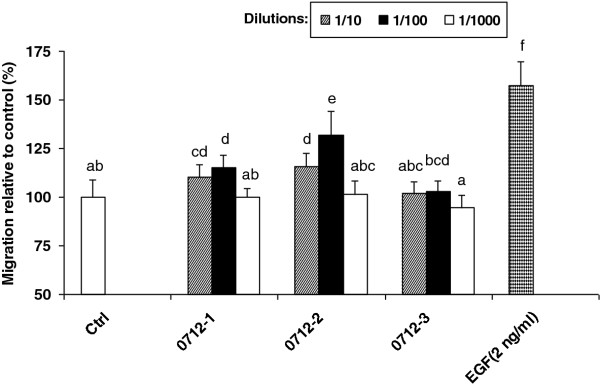
**Effect of substances on fibroblasts migration.** Migration of NIH 3T3 cells (30000/filter) by 0712-1(succussed solvent), 0712–2 (remedy) and 0712–3 (unsuccussed solvent) after 24 h was measured by chemotaxis using 10% FCS as chemoatractant and are expressed as percentages normalized to the untreated control value. As positive control 2 ng/ml EGF was used. Means ± SD from three independent experiments performed in duplicates are presented. All values with different letters are statistically different (p < 0.05).

The proliferative phase is characterized by fibroblast migration followed by angiogenesis and re-epithelization. Cell migration is a process that is essential for tissue repair. Fibroblasts play a key role in dermal wound repair, since they have the ability to migrate and close wounds [2]. For studying the migration and wound healing activity we used the *in vitro* skin equivalent model on an established cell lines mouse NIH 3T3 [19,27]. Soluble growth factors are essential for the regulation of cellular events involved in wound healing, i.e. *inter alia* migration [28]. Chemotaxis was measured using directional fibroblasts migration toward 10% FCS as chemoattractant in modified Boyden chamber. EGF (2 ng/ml) was chosen as positive control, which exerted a preferential effect on cell migration, rarely accompanied by any effect of cell proliferation [29]. Both preparation 0712–1 and 0712–2 stimulated cell locomotion as shown in Figure 1. In comparison to untreated control a significant difference with 0712–1 (p < 0.01) and 0712–2 (p < 0.001) at a dilution of 1:100 was observed in three independent experiments. Migration of cells was stimulated by 14.7% with 0712–1 and 31.9% with 0712–2. Absorbance value of untreated control (1.092) was elevated to 1.253 and 1.440 by substances 0712–1 and 0712–2, respectively. Substances at 1:10 dilution caused an enhancement of 10.5% (0712–1) to absorbance value 1.207 and 15.5% (0712–2) to an absorbance value of 1.262. The highest dilution (1:1000) of both substances did not exert any effect on cell migration. NIH 3T3 cell motility was not influenced by any dilution of ethanol control 0712–3. A negligible elevation of 6.3% was measured with absorbance value of 1.161. EGF accelerated migration of cells by 57.5% to a 1.720 absorbance value. The differences between substance 0712–1 and 0712–2 showed statistical significance for the dilution 1:100 (p < 0.001). Furthermore, remedy (0712–2) differed from 0712–3 significantly for the dilutions 1:10 and 1:100 (p < 0.01 and p < 0.001, respectively). It was surprising, that the substance 0712–1 (succussed solvent) caused a modest enhancement on cell motility, but it was already observed that a succussed solvent exerted biological effects [30]. In spite that we did not find any proliferation effect of substances 0712–1 and 0712–2, the NIH 3T3 cells exposed to these substances showed an increasing migration. It was reported that the migration-promoting activity differs from growth–promoting activity [31] and e.g. EGF caused acceleration of cell migration, without an effect on proliferation rate [29]. Similarly, extracts from *Hypericum perforatum* showed wound healing effect related to its promoting effect on 3T3 fibroblast migration without affecting the cell growth [10]. Cellular proliferation response may not accurately reflect the overall wound healing response. The results of increased cell migration by substances provided confidence for the wound healing experiments.

### Effectiveness of substances on wound closure

**Figure 2:**
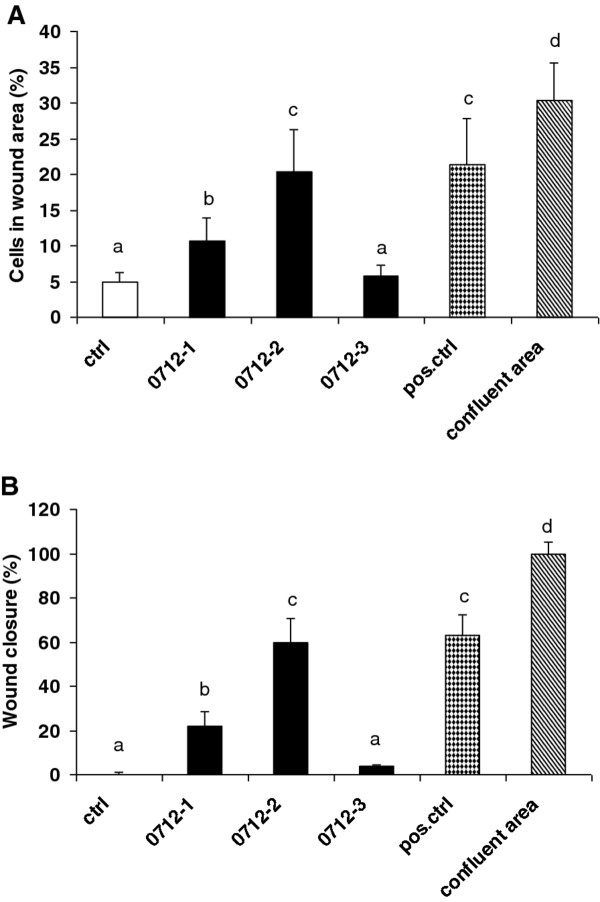
**Wound closure effect of substances.** Effect of substances 0712-1(succussed solvent), 0712–2 (remedy) and 0712–3 (unsuccussed solvent) on the wound closure of NIH 3T3 fibroblasts (25000/well) after 24 h expressed in % of cells migrated into the wound area (**A**) and as percentages of wound closure (**B**). As positive control DMEM with 5% FCS was used. As 100% wound closure the density of cell without created wound was set. Means ± SD of three independent experiments are presented. All values with different letters are statistically different (p < 0.01).

The most important clinical endpoint in wound management is wound closure or 100% epithelization. We used the *in vitro* wound-healing scratch assay in NIH 3T3 fibroblasts which mimics cell migration during wound healing *in vivo*. Specifically, this model assessed cellular wound fill, the “net effect” of all cellular events contributing to the *in vitro* wound healing process, and has been proven as a valuable tool to obtain first insights into how preparations can positively influence the wound closure [8,19]. Further we used a kit, which overcomes the disadvantage of common scratch wound assays lacking a defined wound area by providing proprietary treated inserts that generate a defined wound field. After the wound field was created, NIH 3T3 cells were exposed for 24 h to succussed solvent (0712–1), remedy (0712–2) and unsuccussed solvent (0712–3) in a dilution of 1:100 and 1:1000. As positive control we used DMEM with 5% FCS, because the density of cells was too high at 10% FCS. Migration of cells into the wound was compared to untreated control in DMEM with 2% FCS. Only 4.9 ± 1.3% migrated into the wounded area after 24 h in comparison to time zero (Figure 2A). The level of cellular fill within the wound area in response to substances was compared to the wound-fill response in the presence of 2% FCS as negative control. Density of cells in the controls without a wound (confluent area) was 30.3 ± 5.3% and was set as 100% wound closure. Percentage of cells in the wound area was 10.6 ± 3.4, 20.4 ± 6.0 and 5.7 ± 1.6 for 0712–1, 0712–2 and 0712–3, respectively (Figure 2A). In wound field of positive control (5% FCS) were 21.4 ± 6.4% cells. Substances 0712–1 (succussed solvent) as well as 0712–2 (remedy) exerted significant effects (p < 0.001) and closed the wound to 22.1 ± 6.4% and 59.5 ± 11.4%. The level of wound closure by remedy (0712–2) was about twice the value of succussed solvent (0712–1). This difference in the wound filling effect between 0712–1 and 0712–2 was significant (p < 0.001). However, unsuccussed solvent (0712–3) filled the wound only by 3.7 ± 0.8%. Positive control 5% FCS caused a 63 ± 9.5% wound closure (Figure 2B).

**Figure 3:**
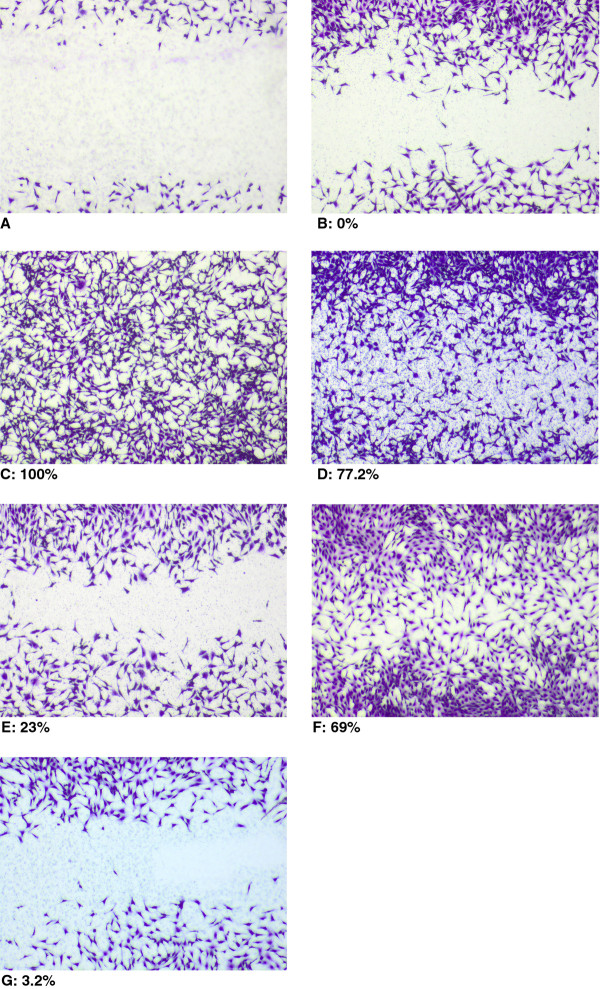
**Light microscope images of the wound closure *in vitro *using confluent monolayer of NIH3T3fibroblasts.** Microphotographs showing one representative experiment of the cell migration into the created wound area in response to the treatment. (**A**) Wound area immediately after wounding and (**B**) after 24 h for the untreated control (medium only, set to 0%); (**C**) confluent area without wounding (set to 100%) as well as treated areas with substances at 1:100 dilution: succussed solvent 0712–1 (**E**), remedy 0712–2 (**F**) and unsuccussed solvent 0712–3 (**G**) after 24 h incubation. DMEM with 5% FCS (**D**) was used as positive control. Wound closure (indicated in%) was normalized to the untreated control (B) and the confluent area (C).

The effect of substances 0712–1, 0712–2 and 0712–3 on the closure of wounded area was investigated only in dilutions of 1:100 and 1:1000, because of the possible influence of 2.2% ethanol at 1:10 dilution. All three substances diluted 1:1000 exhibited only a negligible effect on wound filling, being between 4.2% and 6.3% (data not shown). One representative set of microphotographs on the wound healing effect of substances from three independent experiments is shown in Figure 3. In the chemotaxis migration assay as well as in the wound closure assay, succussed solvent (0712–1) showed a promoting effect on the closure of wound field. The investigated remedy (0712–2) filled the gap between the cells comparable to the positive control (5% FCS) and we could establish its promoting effect on wound closure in comparison to the reduced fill rate of the control.

*In vivo* effectiveness of low potency homeopathic remedies containing arnica, marigold, St. John’s wort or comfrey on wound healing has been reported in humans [14,15]. However, experimental studies were mainly based on animal models [32-34]. The concentrations of homeopathic remedies in the above mentioned studies ranged from 1x, 4x, 12x, 5c, 6c up to 1 M. Homeopathic remedies of *Calendula* and *Hypericum* applied were in the range of mother tincture (1x) in a rat model [33], but even *Arnica* 12x showed positive influence on wound healing in rats [32]. In one trial Arnica 4x (10 pills, 3 times per day) was equivalent to diclofenac (50 mg, 3 times per day) for wound irritation yet, not pain reduction after foot surgery [15]. Patients taking perioperative homeopathic *Arnica montana* (5c-1 M) exhibited statistically significant less postoperative ecchymosis compared to placebo in a double blind clinical trial [14], but this effect could not be confirmed in a double blind trial conducted by others [17]. According to the available literature in medical databases the wound-healing effect of homeopathic remedies in *in vitro* models are scare or lacking. Bressler et al who studied the effect of *Calendula officinalis* 3c and low level laser therapy on wound healing in human skin fibroblasts described an accelerating effect on wound closure and increased cell viability by *Calendula*. Effective skin penetration ability of a remedy is an important factor for topical response and wound healing. It was reported that low concentrated *Arnica* preparations increased permeation through porcine skin [35] as well as human skin *in vitro*[36].

Several natural products have been shown to effectively accelerate wound healing [7] at pharmacological concentrations. The active constituents of these plants are mainly flavonoids, polyphenols, sesquiterpenes, essential oils, and tannins among other constituents [2]. The antioxidant, antiinflammatory effects exerted may be attributed to their wound healing effectiveness [37-39]. A mother tincture from *Arnica montana* exerted inhibition of 5-lipoxygenase/cyclooxygenase in *in vitro* experiments [40] and even at concentrations of 6c [13] and 4x [34] anti-inflammatory activity was shown in the carrageenan-induced rat paw oedema. Therefore the question of concern is whether low potency homeopathic remedies can exert biological effects in experimental cell models. This hypothesis is in line with the findings of our study where the final effective wound fillling concentrations were 289 ng/ml of *Arnica montana*, 28.9 ng/ml *Calendula officinalis*, 89.4 ng/ml *Hypericum perforatum* and 0.823 ng/ml *Symphytum officinalis* expressed in dry weight of single herbs in the examined remedy (0712–2). It has been reported, that compounds at high dilutions/low concentrations could exert different biological activity. TNF-α up to 100x from 100 ng/ml elevated the level of H_2_O_2_ in SK-N-SH neuroblastoma cells [30], arsenic of decimal and centesimal dilutions exerted effect in the rats [41], histamine dilutions ranging between 15-19c from 1 mg/ml inhibited human basophil degranulation [42]. In addition, normal and human leukemia T-lymphocytes responded to cadmium chloride at low doses (nM-μM; 0.2-200 ng/ml) [43]. Based on this it could be speculated, that the wound closure effect of the homeopathic remedy 0712–2 in NIH 3T3 fibroblasts may be due to the exerted properties of active ingredients at low concentrations.

In experiments with homeopathic preparations difficulties with the reproducibility even of *in vitro* models are known [44]. The present findings need to be confirmed in further studies before the chemotaxis and wound closure (scratch) model can be used to investigate various questions of interest in the *in vitro* research of homeopathic remedies. In the present study we described the *in vitro* wound closure effect of preparation 0712–2 on one cell type (NIH 3T3 fibroblasts) involved in the overall wound healing process. Final proof as wound healing remedy can only be done by *in vivo* studies.

## Conclusions

In this study we showed (i) that the *in vitro* wound model used was sensitive enough to observe effects of substances at low potency homeopathic concentrations and therefore could be further exploited for the development of an useful *in vitro* model.

We (ii) investigated the contribution of proliferation and migration towards the resulting wound fill by the remedy (0712–2). Its promoting wound filling effect could be related to the increased cell migration without an increased mitotic activity of cells.

## Competing interests

The authors declare that they have no competing interests.

## Authors’ contributions

KH and RS are responsible for the study design, analysis and data interpretation as well as the manuscript preparation. KH conducted the assays and analyses. SB performed the statistical evaluations and helped with the draft of manuscript. MR and JM participated in data analysis and drafted the manuscript. All authors read and approved the final manuscript.

## Pre-publication history

The pre-publication history for this paper can be accessed here:

http://www.biomedcentral.com/1472-6882/12/100/prepub
